# Anti-Tick-Borne Encephalitis Virus Activity of Novel Uridine Glycoconjugates Containing Amide or/and 1,2,3-Triazole Moiety in the Linker Structure

**DOI:** 10.3390/ph13120460

**Published:** 2020-12-13

**Authors:** Gabriela Brzuska, Gabriela Pastuch-Gawolek, Monika Krawczyk, Boguslaw Szewczyk, Ewelina Krol

**Affiliations:** 1 Department of Recombinant Vaccines, Intercollegiate Faculty of Biotechnology, University of Gdansk and Medical University of Gdansk, Abrahama 58, 80-307 Gdansk, Poland; gabriela.brzuska@phdstud.ug.edu.pl (G.B.); boguslaw.szewczyk@biotech.ug.edu.pl (B.S.); 2 Department of Organic Chemistry, Bioorganic Chemistry and Biotechnology, Faculty of Chemistry, Silesian University of Technology, Krzywoustego 4, 44-100 Gliwice, Poland; gabriela.pastuch@polsl.pl (G.P.-G.); monika.krawczyk@polsl.pl (M.K.); 3 Biotechnology Centre, Silesian University of Technology, Krzywoustego 8, 44-100 Gliwice, Poland

**Keywords:** uridine glycoconjugates, antiviral activity, glycosyltransferase inhibitors, flavivirus, tick-borne encephalitis virus

## Abstract

Tick-borne encephalitis virus (TBEV) transmitted by ticks is a pathogen of great medical importance. As still no effective antiviral treatment is available, in the present study, a series of uridine glycoconjugates containing amide or/and 1,2,3-triazole moiety in the linker structure was synthesized and evaluated for the antiviral activity against two strains of TBEV: a highly virulent Hypr strain and less virulent Neudoerfl strain, using standardized previously in vitro assays. Our data have shown that four compounds from the series (**18–21**) possess strong activity against both TBEV strains. The half maximal inhibitory concentration (IC_50_) values of compounds **18–21** were between 15.1 and 3.7 μM depending on the virus strain, which along with low cytotoxicity resulted in high values of the selectivity index (SI). The obtained results suggest that these compounds may be promising candidates for further development of new therapies against flaviviruses.

## 1. Introduction

As the present pandemic situation shows, emerging viral infections cause many adverse effects, not only directly on human health, but also on all aspects of our lives. One of such potential dangers of viral origin are flaviviruses possessing positive-sense RNA genomes. Many viruses of the *Flaviviridae* family are associated with human diseases of great medical importance. In the past few decades flaviviruses, due to the climate change, have tremendously widened their range and have longer periods of activity, resulting in over 400 million infections per year, due to which they are a constant threat to public health [1]. Flaviviruses infect not only humans, but also many other animal hosts, like birds, rodents, non-human primates and many other mammals. Their transmission is highly correlated with the expansion of the geographical range of their insect vectors—ticks and mosquitos. Recently, many epidemics and outbreaks of emerging and re-emerging flaviviruses (such as Zika virus (ZIKV), dengue virus (DENV), yellow fever virus (YFV) or West Nile virus (WNV)) have been observed, which has raised the importance of the research concerning treatment and prevention methods [2,3,4].

Within the group of tick-transmitted viruses, tick-borne encephalitis virus (TBEV) is one of the most pathogenic flaviviruses affecting the human central nervous system. It causes a seasonal disorder called tick-borne encephalitis (TBE), which may lead to severe and long-lasting complications including meningitis, meningoencephalitis, partial paralysis, coma or even death [5]. TBEV is endemic in over 30 countries of Europe and Asia and new endemic centers are constantly reported [6]. In these regions several TBEV subtypes are circulating, with three main subtypes: European (TBEV-Eu), Siberian (TBEV-Sib), Far Eastern (TBEV-FE) and two newly discovered: Baikalian (TBEV-Bkl) and Himalayan (TBEV-Him) [7,8]. These subtypes differ not only in the geographical distribution, which depends on their tick vector (*Ixodes ricinus*—TBEV-Eu, *Ixodes persulcatus*—TBEV-FE and TBEV-Sib), but most importantly in the severity of the TBE course and fatality rate. A bite of an TBEV infected tick is the most common route of human infection; however, other routes like consumption of unpasteurized milk or milk products from infected ruminants are also possible [9]. Although few vaccines against TBEV are on the market, vaccination is not mandatory in most of the affected countries resulting in around 13,000 annual cases worldwide [6,10]. Moreover, up to this date no effective and licensed treatment for TBEV infection is available, although different antiviral compounds are being tested in the laboratories [11,12,13,14]. For the treatment of infected patients, the only strategy being implemented in hospitals is to alleviate the symptoms. In light of these facts, the research on the new antiviral drugs is highly justified.

From the structural point of view, members of the *Flavivirus* genus share several morphological similarities. TBEV is a small, enveloped virus with a mean diameter of 50 nm. The genome, surrounded by the capsid (C) is in the form of a ssRNA (+), which encodes a single polyprotein cleaved to three structural: C, prM/M, E and five non-structural proteins (NS1–5) [15]. The prM/M and E proteins are anchored in the envelope of the virus. The role of the E protein in the TBEV cycle is well characterized. It mediates entry via receptor mediated endocytosis, and contains the major antigenic epitopes used for generation of protective immune response [16]. Three of the flaviviral proteins usually possess at least one *N*-glycosylation site: prM, E and NS1 and these proteins play a crucial role during the virion assembly and maturation, entry to the cells, immune evasion and replication. The role of glycans in the flavivirus replication cycle is not fully understood. For TBEV, it has been shown that removal of the glycosylation site in the E protein leads to the reduction in infectivity of secreted virions [17]. Therefore, it is important to examine the role of glycans in each flavivirus infection separately and to test the activity of glycosylation inhibitors as potential antiviral drugs.

Various glycosyltransferases (GT) may be utilized by flaviviruses to attach specific glycans onto their proteins. Inhibition of one of the GT with synthetic compounds may be a promising strategy for antiviral treatment for different flaviviruses. Previously, we have shown the antiviral activity of different uridine derivatives targeting GT against members of the *Flaviviridae* family—classical swine fever virus (CSFV), hepatitis C virus (HCV), and also against TBEV [18,19,20]. Moreover, the activity of these compounds was tested against influenza virus (*Orthomyxoviridae* family member) [21]. The inhibitory effect of these compounds was associated with the impaired maturation and secretion of infectious viruses. These studies showed that GT inhibitors could be used as broad-spectrum antiviral drugs against different human and animal viruses.

In this study, we present the design, synthesis and antiviral activity of a novel type of uridine glycoconjugates, containing amide or/and 1,2,3-triazole moiety in the linker structure, against two strains of tick-borne encephalitis virus, a main cause of human neuroinfections.

## 2. Results

### 2.1. Synthesis of Glycoconjugates **13**–**23**

**Figure 1:**
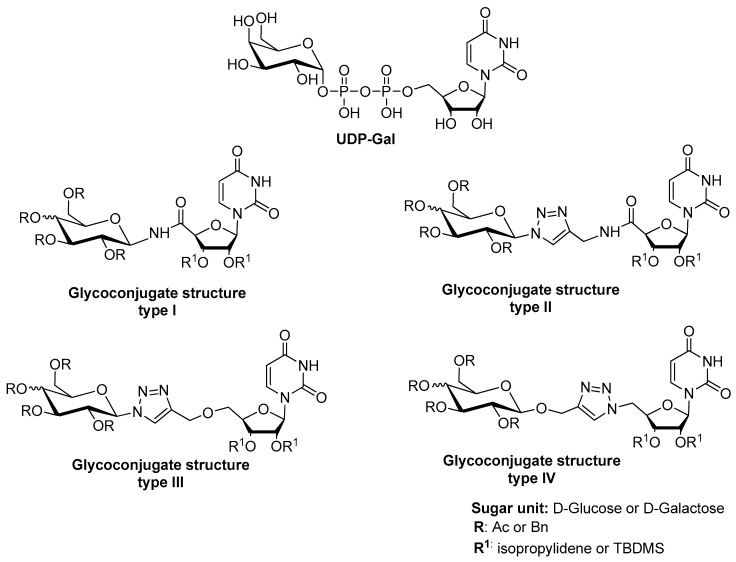
The general structures of glycoconjugates, analogues of the natural β4GalT substrate—UDP-Gal.

We designed compounds that can compete with natural glycosyltransferase substrates for sites in their active centers. As previously published, natural donor-type substrates contain in their structure a sugar unit linked via a diphosphate moiety to uridine [22,23]. Conventional modification methods of glycosyltransferase inhibitors may include the replacement of the diphosphate moiety and connection of the sugar unit via a non-charged linker to the uridine part. Such a linker should be capable of coordinating the divalent metal ion (usually magnesium or manganese) bound in the active site of the enzyme. In the tested glycoconjugates, the diphosphate moiety was replaced with an amide linker between carbohydrate and uridine moiety (glycoconjugate structure type I, Figure 1) or with a methylene 1,2,3-triazolyl part linked to a sugar or uridine moiety via an amide or ether linkage (glycoconjugate structures types II-IV, Figure 1). Recently, the results of studies showing that a completely deprotected type IV glycoconjugate is able to inhibit the activity of β-1,4-galactosyltransferase I (β4GalT) were reported [24]. There are also reports of the synthesis of a (triazolyl) methyl amide-linked disaccharide nucleosides as analogues of nucleoside diphosphate sugars. Some of the obtained 6′-isonucleosides and triazole-containing glycoderivatives displayed acetylcholinesterase inhibitory activities [25]. Nucleoside triphosphate mimetic, where the phosphate residues-containing chain was replaced by an uncharged methylene-triazole moiety, are also known to have occurred. These compounds have been evaluated as competitive inhibitors of *Bacillus anthracis* pantothenate kinase [26]. Therefore, it can be assumed that the 1,2,3-triazole ring in the linker structure is an important element for glycoconjugate activity. The aim of the present work was to investigate the influence of the linker structure containing the 1,2,3-triazole system on the biological activity of the obtained connections. Glycoconjugates structure type III have the reverse direction of linking reactive moieties (as a result of the reaction between sugar azides and 5′-propargyl uridine derivative) compared to the glycoconjugate structure type IV. In turn, in the glycoconjugate structure of type II, an additional amide moiety was introduced between the methylene 1,2,3-triazolyl part and uridine compared to the structure type III. It was presumed that insertion into a linker of an additional amide bond, the presence of which is observed in many biologically active compounds should improve glycoconjugates’ chelating ability, and thus their binding ability at the metal-dependent enzyme active center [27].

The key reactions to obtain the planned glycoconjugate structures are the condensations between the amine and carboxyl derivatives leading to the formation of an amide bond as well as copper-catalyzed 1,3-dipolar azido-alkyne cycloaddition to form a 1,2,3-triazole system. Therefore, it was necessary to prepare appropriate sugar and uridine derivatives, which were then be used in the synthesis of glycoconjugates.

**Scheme 1:**
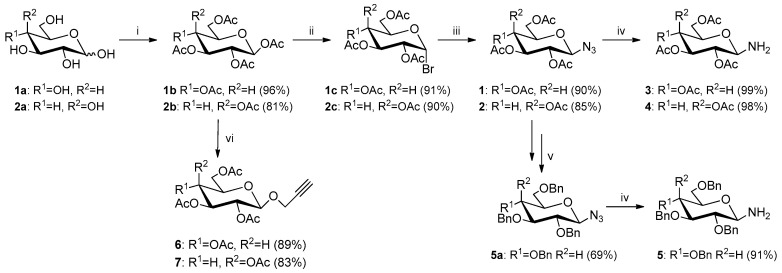
Synthesis of sugar derivatives. Reagents and conditions: (i) CH_3_COONa, Ac_2_O, b.p., 1 h; (ii) CH_3_COOH, 33% HBr/AcOH, r.t. 1 h; (iii) NaN_3_, TBASH, CHCl_3_/NaHCO_3_, r.t. 2 h; (iv) 20% Pd(OH)_2_/C, THF:EtOH (2:1, *v*/*v*), H_2_, 1.5 bar, 2 h; (v) 1. NaOMe, MeOH, r.t. 20 min; 2. Amberlyst-15, 3. BnBr, NaH, DMF, r.t., 24 h; (vi) propargyl alcohol, BF_3_·Et_2_O, CH_2_Cl_2_, r.t., 1 h.

The first part of the synthesis was devoted to the sugar building blocks necessary for glycoconjugates synthesis. The synthetic pathways leading to the formation of sugar derivatives are presented in Scheme 1. All substrates were prepared according to the previously published procedures involving the acetylation of free sugars **1a** or **2a** and conversion of per-*O*-acetylated derivatives **1b** or **2b** into the corresponding glycosyl bromides **1c** or **2c** [22,28]. The glycosyl bromides were used immediately for further reactions leading to obtain 2,3,4,6-tetra-*O*-acetyl-β-glycosyl azides **1** and **2** [23]. To obtain glucosyl azide **5a** with benzyl protecting groups, it was necessary to remove the acetyls from compound **1** under Zemplén conditions [29] which allowed the attainment of deprotected β-glycosyl azide which was benzylated with benzyl bromide in the presence of sodium hydride in anhydrous DMF [30]. β-D-Glycopyranosyl amines **3–5** were obtained from the corresponding glycosyl azides **1**, **2** or **5a** through a hydrogenation reaction in a Parr apparatus using palladium hydroxide deposited on activated carbon [31].

Sugar derivatives **6** and **7** in which the alkynyl moiety was introduced were prepared by reacting per-*O*-acetylated D-glucose **1a** or D-galactose **1b** with propargyl alcohol in the presence of a Lewis acid as a catalyst [32]. The acetyl neighboring-group participation at the C-2 position of the sugar ensured the formation of only products of the β-configuration. As a result, propargyl 2,3,4,6-tetra-*O*-acetyl-β-D-glycopyranosides **6** and **7** were obtained with good yields (89% and 83%, respectively). 

**Scheme 2:**
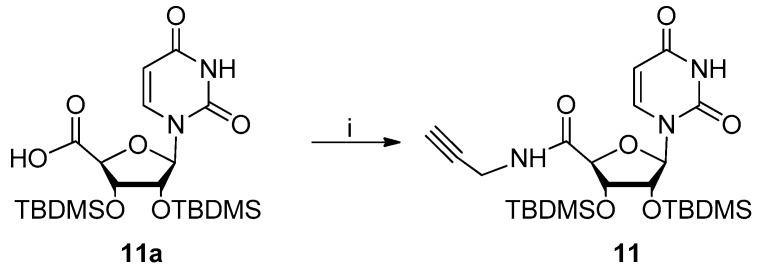
Synthesis of uridine derivative **11**. Reagents and conditions: (i) propargylamine, DCC, CH_2_Cl_2_, r.t, 24 h.

The synthesis of uridine derivatives **8–10**, a second component necessary for the construction of glycoconjugates, as well as compounds **11a** and **12a** were described in the earlier works of our group [18,24]. The counterpart of uridine derivative **10** with TBDMS-protecting groups was obtained by adding propargyl amine to the compound **11a** using DCC as an amide coupling agent (Scheme 2) [33]. The product **11** was obtained in the form of a colorless oil with a yield of 75%.

**Scheme 3:**
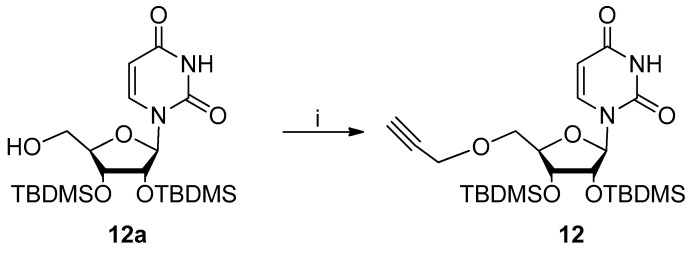
Synthesis of uridine derivative **12**. Reagents and Conditions: (i) propargyl bromide, NaH, THF, r.t., ultrasonic bath, 4 h.

The last uridine derivative was obtained by reacting compound **12a** with propargyl bromide in anhydrous tetrahydrofuran using sodium anhydride as a base (Scheme 3). Carrying out the reaction in an ultrasonic bath increases the contact surface of the reactants, and thus accelerates the reaction. Thanks to the procedure used, the product **12** was obtained with a yield of 59%.

**Scheme 4:**
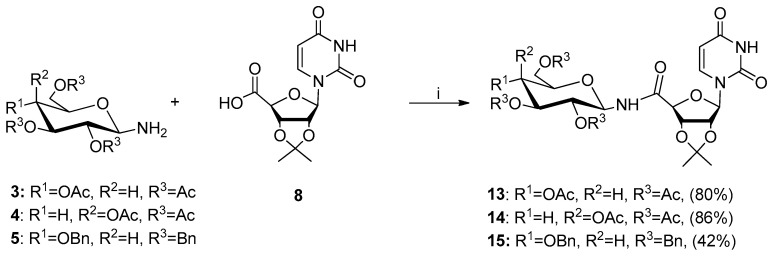
Synthesis of glycoconjugates type I. Reagents and conditions: (i) CDMT, NMM, MeOH, r.t., 5 h.

The synthesis of glycoconjugates was started with glycoconjugates **13–15** which were obtained in the condensation reaction between 1-aminosugars **3–5** and uridine derivative **8** as an acid donor, using generated in situ an amide coupling agent (4-(4,6-dimethoxy[1,3,5]triazin-2-yl)-4-methylmorpholinium chloride) (DMTMM). The procedure worked well for glycoconjugates **13** and **14** obtained from acetyl protected sugar derivatives (80% and 86% yield respectively, Scheme 4), however in the case of 1-aminosugar with benzyl protection, it was possible to obtain glycoconjugate **15** only with a moderate yield (42%). A possible explanation for this may be the excessively high reactivity of the amino sugar with electron-donating protecting groups, which leads to the formation, in addition to the desired glycoconjugate, also of by-products. Indeed, the spots, not only from the formed glycoconjugate, but also from other sugar-containing products were observed on the thin layer chromatography (TLC) plates used to follow the reaction progress.

**Scheme 5:**
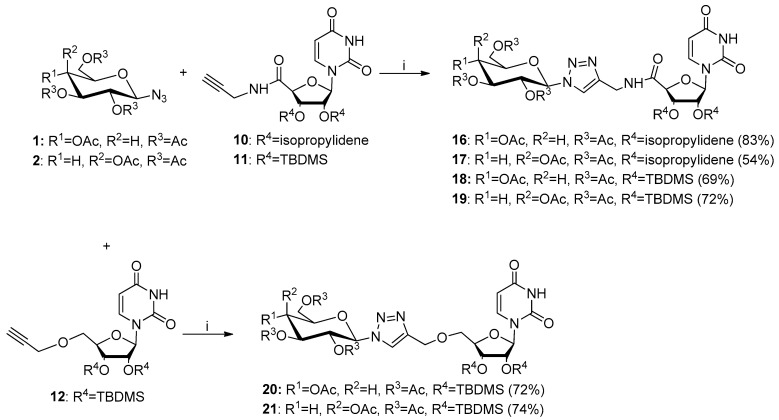
Synthesis of glycoconjugates type II and type III. Reagents and Conditions: (i) CuSO_4_·5H_2_O, NaASC, THF/*i*-PrOH, H_2_O, r.t.

Other glycoconjugates, which were assumed to have a 1,2,3-triazole system in the linker structure between the sugar fragment and uridine, were obtained using copper-catalyzed azide-alkyne 1,3-dipolar cycloaddition (CuAAC) [34,35]. For the preparation of glycoconjugates **16–19** CuAAC reactions between 1-azido sugars **1** or **2** and uridine derivatives **10** or **11** were performed (Scheme 5). As the result of the reactions, glycoconjugates **16–19** were obtained with good yields. In order to be able to assess whether the amide bond introduced into the linker structure has a significant influence on the biological activity of the final compounds, glycoconjugates **20** and **21** were prepared in parallel in which the amide bond in the uridine fragment has been replaced with a linkage via an oxygen atom.

**Scheme 6:**
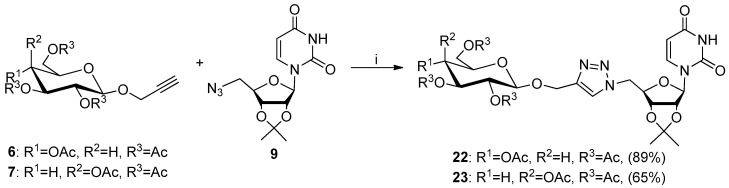
Synthesis of glycoconjugates type IV. Reagents and Conditions: (i) CuSO_4_·5H_2_O, NaASC, THF/*i*-PrOH, H_2_O, r.t.

The last group of type IV glycoconjugates was obtained in order to check whether the reverse orientation of the 1,2,3-triazole system in the linker structure has a significant influence on the biological activity of these connections. In case of the synthesis of glycoconjugates **22** and **23**, propargyl glycosides **6** and **7** and the uridine derivative **9** containing an azide moiety in the C-5′ position were used as substrates for the CuAAC reaction (Scheme 6).

All the carried out CuAAC reactions proceeded with good or very good yields and led to the formation of only derivatives containing 1,4-disubstituted 1,2,3-triazole. The structures of all glycoconjugates were confirmed by means of nuclear magnetic resonance (NMR) and high resolution mass spectroscopy (HRMS.)

### 2.2. Biological Evaluation

#### 2.2.1. Antiviral Activity of Uridine Glycoconjugates Against Tick-Borne Encephalitis Virus

As the initial steps of the biological evaluation of synthesized compounds we performed cytotoxicity studies according to the established method [19]. Cytotoxicity was analyzed using MTS-based cell proliferation assay in the permissive for TBEV—A549 cell line. Cytotoxic concentration (CC_50_) values for each compound were estimated in order to choose non-toxic doses for further antiviral activity studies. CC_50_ values for the compounds **13–23** were 129, 114, 153, 136, 137, 69, 77, 184, 116, 173 and 157 µM, respectively. All of the uridine glycoconjugates showed dose-dependent cytotoxic effects with relatively moderate level of toxicity; below a concentration of 25 µM all of the compounds did not significantly influence the viability of the cells.

**Figure 2:**
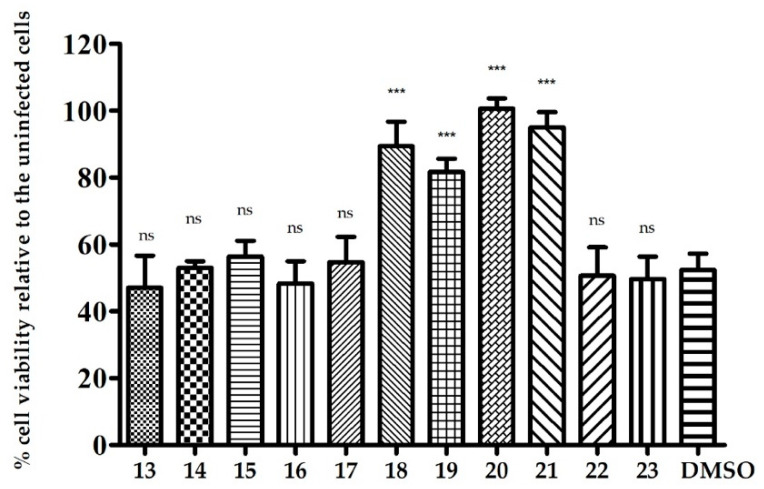
The effect of uridine glycoconjugates on tick-borne encephalitis virus (TBEV)-induced cytopathic effect. A549-infected cells (TBEV Hypr strain (multiplicity of infection (MOI) of 0.1)) were treated with 25 µM of tested compounds or DMSO. After 48 h, the cell death was measured and calculated as the percentage of the cell viability. Error bars represent standard deviations from 3 separate experiments. ns: not significant; ***: *p* < 0.001 compared to infected DMSO-treated cells by one-way ANOVA with Dunnett’s post-test.

Preliminary screening of antiviral effects of eleven tested compounds was performed using two TBEV strains (Hypr and Neudoerfl) by assessing cytopathic effect (CPE) inhibition assay and plaque reduction assay as previously reported [19]. The compounds were tested at the non-toxic concentration of 25 µM. TBEV Hypr strain is a highly cytopathic virus causing A549 cell death 96 h post-infection. To analyze the antiviral activity of the tested compounds, their protective effect on cell survival was measured using a colorimetric assay. In A549-TBEV infected DMSO-treated cells the rate of cell death was about 50% (Figure 2). In A549-TBEV, infected cells treated with 25 µM of compounds **18**, **19**, **20** and **21**, the rate of cell death was significantly decreased. Two compounds (**20** and **21**) were the most active from the series. TBEV-induced CPE effect was nearly completely inhibited after treatment with 25 µM of both compounds. The cell death was also strongly reduced after treatment with two other compounds (**18** and **19**). The inhibition of cell death was not observed for other tested compounds. A strong CPE was still observed in A549 cells treated with these compounds, indicating that these compounds have almost no antiviral activity against TBEV.

**Figure 3:**
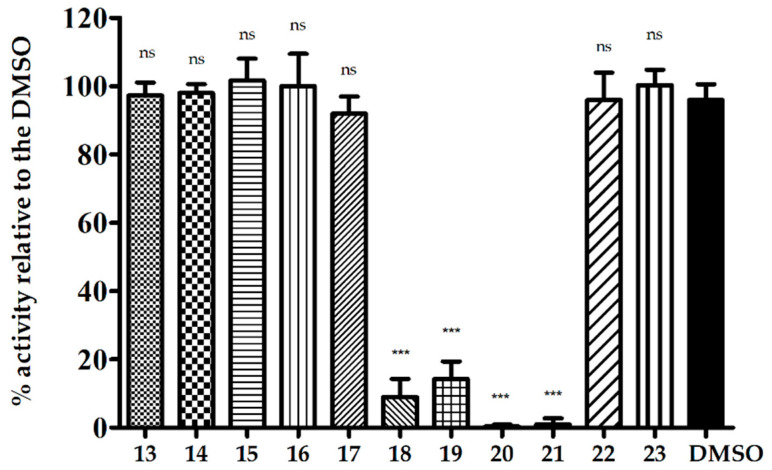
The effect of uridine glycoconjugates on TBEV infection in A549 cells. A549 cells were infected with the TBEV Neudoerfl strain at an MOI of 0.001. At 2 h post-infection (p.i.) the virus was removed, cells were washed and overlaid with carboxymethylcellulose in Dulbecco’s Modified Eagle’s Medium with DMSO or various doses of tested compounds. At 48 h p.i., cells were fixed, and infected foci were visualized by immunostaining with the monoclonal anti-Flavivirus group antigen antibody (4G2). Viral plaques were counted and expressed as percentage in comparison to the number of plaques detected in DMSO-treated infected cells set as 100%. Error bars represent standard deviations from 3 separate experiments. ns: not significant; ***: *p* < 0.001 compared to infected DMSO-treated cells by one-way ANOVA with Dunnett’s post-test.

These results were further confirmed using plaque reduction assay where the dose of 25 µM of all synthesized compounds was tested for the inhibition of TBEV propagation in A549 cells using the immunoperoxidase monolayer assay (IPMA). A low multiplicity of infection (0.001) of low cytopathic Neudoerfl strain was used to detect the single areas of infected cells by the immunostaining of E protein. The infected DMSO-treated cells were used as positive control. Viral plaques were counted and expressed as percentage in comparison to the number of plaques detected in the positive control set as 100%. As shown in Figure 3 compounds **18**, **19**, **20** and **21** effectively inhibited virus propagation in comparison to infected DMSO-treated cells. The reduction in size and number of plaques after 48 h compounds treatment was observed. As in previous experiment compounds **20** and **21** were the most active causing nearly a complete inhibition of TBEV propagation. Treatment with compounds **13–17** and **22–23** had no effect on TBEV infection; therefore, these compounds were not used in further analysis.

#### 2.2.2. Dose-Response Activity of Uridine Glycoconjugates Against Tick-Borne Encephalitis Virus

**Figure 4:**
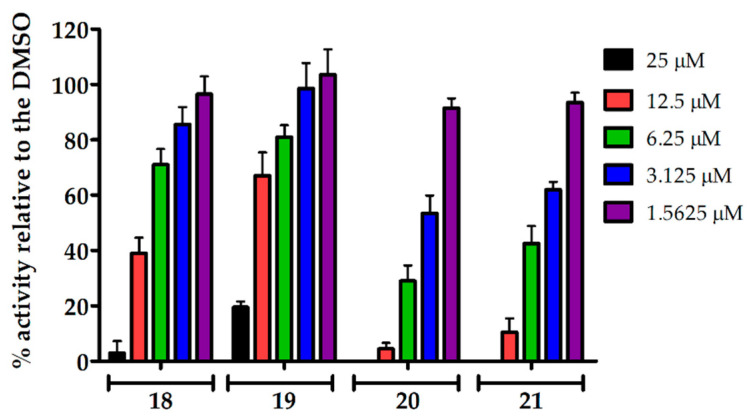
The effect of uridine glycoconjugates on TBEV infection in A549 cells. A549 cells were infected with the TBEV Neudoerfl strain at an MOI of 0.001. At 2 h p.i. the virus was removed, cells were washed and overlaid with carboxymethylcellulose in Dulbecco’s Modified Eagle’s Medium with DMSO or various doses of tested compounds (0–25 µM). At 48 h p.i., cells were fixed, and infected foci were visualized by immunostaining with the monoclonal anti-Flavivirus group antigen antibody (4G2). Viral plaques were counted and expressed as percentage in comparison to the number of plaques detected in DMSO-treated infected cells set as 100%. Error bars represent standard deviations from 3 separate experiments.

Four compounds that showed strong TBEV antiviral activity in the initial experiments were further evaluated to determine the dose-response inhibitory effect. Neudoerfl TBEV-infected monolayers of A549 cells (multiplicity of infection (MOI) = 0.001) were incubated with increasing concentrations of the most active compounds: **18, 19, 20, 21** (0–25 µM) diluted in the overlay medium for 48 h and the plaque reduction assay described above was performed. Plaques were immunostained and counted. All four tested compounds showed a dose-dependent reduction in the number of positive infected foci (Figure 4). Compounds **20** and **21** were the most active because the highest inhibition of plaque formation was observed in the case of all tested doses of these compounds. At the dose of 25 µM no plaques were detected. The dose of 12.5 µM of both compounds significantly reduced the number of plaques by 96% and 92%, respectively, when compared to infected DMSO-treated cells. Moreover, the dose of 6.25 µM caused 75% and 62% reductions in the number of plaques for compounds **20** and **21**, respectively. For less active compounds **18** and **19**, the decrease in the number of plaques at the highest doses of 25 and 12.5 µM was also observed. An amount of 25 µM of both compounds reduced the number of plaques by 98% and 81%, respectively.

Next, compounds **18–21** have been tested for their effect on the infectious virus titers in a dose-response assay. A549 cells were infected with low MOI of the virus (0.001, Neudoerfl strain) and treated with 0–25 µM concentrations of compounds. The culture supernatants collected 72 h p.i. were used to determine the viral titers using the plaque assay. The results were used to determine half-maximal inhibitory concentration (IC_50_) values and to calculate selectivity index (SI) values (CC_50_/IC_50_ ratio) shown in Table 1.

**Figure 5:**
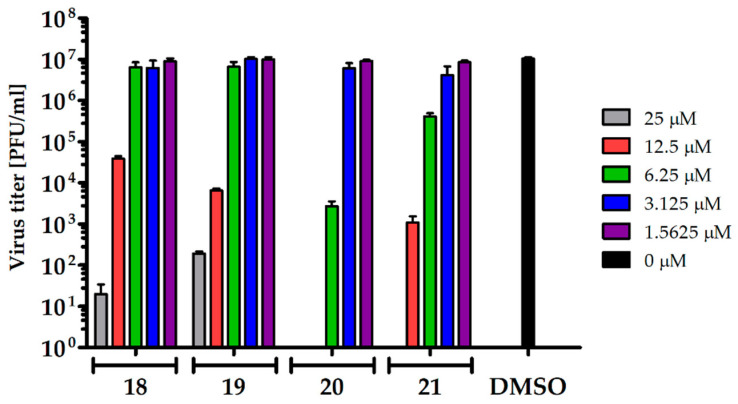
The effect of uridine glycoconjugates **18**, **19**, **20** and **21** on TBEV titers. A549 cells were infected with the TBEV Neudoerfl strain at an MOI of 0.1 and treated with various doses of tested compounds (0–25 µM). At 72 h p.i., culture supernatants were collected to determine TBEV titers by plaque assay. Error bars represent standard deviations from 3 separate experiments.

The titer for the positive control (TBEV collected from non-treated cells) was 1 × 10^7^ plaque-forming unit/mL (PFU/mL). Four tested compounds (**18**, **19**, **20** and **21**) significantly reduced TBEV titers in a dose-dependent manner confirming their anti-TBEV activity (Figure 5). Compounds **20** and **21** were the most active. After treatment with 25 µM of both compounds and 12.5 µM of compound **20** no virus was detected. Treatment with 6.25 µM of compound **20** and 12.5 µM of compound **21** caused 10^3^–10^4^-fold reduction in viral titer, respectively. Compounds **18** and **19** caused nearly 10^3^–10^6^-fold reduction in viral titer after treatment with 25 and 12.5 µM. The highest IC_50_ value—3.7 µM was obtained for compound **20** and due to its low cytotoxicity it reached a high SI of 49.7.

#### 2.2.3. The Activity of Uridine Glycoconjugates on Protein Synthesis

**Figure 6:**
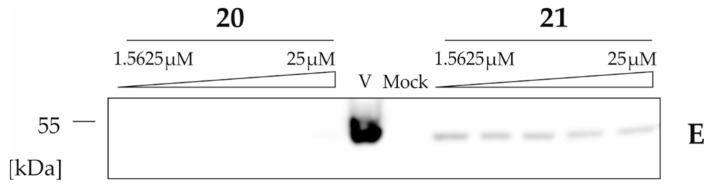
Analysis of TBEV E glycoprotein synthesis after treatment with uridine glycoconjugates. A549 cells were infected with TBEV Neudoerfl strain, MOI of 0,1. At 2 h p.i., cells were treated with various doses of compound **20**, **21** or DMSO diluted in the medium for 48 h. Cell lysates were prepared and separated by SDS-PAGE (gradient 10–20% polyacrylamide). The E protein was detected in Western blot analysis using monoclonal anti-Flavivirus group antigen antibody (4G2).

Previously, we reported that the synthesis of viral glycoproteins of CSFV and influenza virus is impaired after treatment with uridine derivatives [36,37]. The uridine glycoconjugates described in this report were also designed to target glycosyltransferases, therefore we assessed the influence of tested compounds on TBEV glycoprotein production using Western blot analysis of TBEV Neudoerfl strain infected and treated cells lysates. A dose-dependent reduction in the production of E protein was observed after treatment with compounds **18**, **19** and **21**. The representative results for compound **21** are shown in Figure 6. To our surprise, all tested doses of compound **20** (25–1.5625 µM) caused the complete inhibition of viral protein synthesis as E protein was not detected in Western blot analysis (Figure 6). After treatment with each of tested compounds, the non-glycosylated and under-glycosylated form of E protein were not detected probably due to their degradation.

## 3. Discussion

Due to the continuous changes of the global climate, which influences the geographical distribution of ticks and mosquitoes, flaviviruses are an expanding threat to the human population. New endemic centers and outbreaks of emerging and re-emerging flaviviruses are often reported. Although, vaccines against some flaviviruses are commercially available e.g., anti-TBEV and anti-YFV, vaccination is not mandatory, or it is not 100% effective. Moreover, in spite of much effort put in the development of the effective antiviral therapy against flaviviruses, no drugs were approved for clinical use. Tick-borne encephalitis virus causing the most important and serious neurological infections is one of the most pathogenic flaviviruses constituting a public health problem in Europe and Asia. Several thousand cases of TBE are registered annually, however due to the large number of asymptomatic infections the scale is much wider.

*N*-glycans attached to flavivirus proteins play multiple roles in the replication cycle including attachment to the host cell, assembly and secretion of virions. Glycosylated E protein of flaviviruses may interact with cell surface lectins during the attachment step. The interaction of C-type lectin dendritic cell-specific intercellular adhesion molecule-3-grabbing nonintegrin (DC-SIGN) and DC-SIGN-related protein (DC-SIGNR) with E glycoprotein of many flaviviruses: ZIKV, WNV, DENV and Japanese encephalitis virus (JEV) was reported [38,39,40,41,42]. The interaction with DC-SIGN and DC-SIGNR depends on the virus, presence of the glycan as well as on the complexity of the glycan chain. However, these type of lectins are expressed only on the macrophages and dendritic cells (DCs) or on microvascular endothelial cells [43]. Another study showed that glycosylation of ZIKV E protein plays a role during attachment to other cell types like A549 cell line, but is dispensable for infection in Vero cells [44]. Both cell lines lack DC-SIGN or DC-SIGNR, which may suggest that other lectins may be involved in the attachment of virus to some cell types. Moreover, glycosylation of both flavivirus envelope proteins prM and E could be also a crucial factor for proper folding, assembly and egress through the secretory pathway, although involvement of glycans in these processes has not been fully understood. The ablation of the *N*-glycosylation site in the prM or E protein of TBEV, ZIKV or WNV influences the expression and secretion of virus-like particles or subviral particles [45,46,47]. These reports suggest that glycan chains may stabilize formation of E protein dimers or may interact with some chaperone-like proteins during the transport of virions through the secretory compartments. For TBEV it has been shown that inhibition of *N*-glycosylation of E protein or glucose trimming of the carbohydrate side chain of this protein results in the decrease in VLPs secretion, suggesting the role of *N*-glycans in assembly and/or secretion step [48]. In another study, it has been proven that the glycan associated with TBEV E-154 glycosylation site plays an important role in the secretion of VLPs from mammalian cells, however the glycosylation of the prM protein plays a less important role in this process [45]. Moreover, the impairment of TBEV E protein glycosylation did not affect the total level of secreted E protein in mammalian cells, however the conformation structure of the protein was affected resulting in the reduction in infectivity of secreted virions also in a mice model. In contrast, the deletion of the glycosylation site of E protein did not affect TBEV growth in tick cells [17]. The significant difference in the carbohydrate profile of TBEV E protein when grown in human and tick cells can be the explanation of this phenomenon [49]. As glycosylation plays highly crucial, yet multifaceted roles during the flavivirus replication cycle, we anticipate that targeting of this process may be a promising approach to antiviral therapy.

As the first step of our research, we evaluated the antiviral activity of 11 novel uridine glycoconjugates containing amide or/and 1,2,3-triazole moiety in the linker structure against TBEV, a member of the *Flavivirus* genus. At the initial screening, four compounds (**18**, **19**, **20**, **21**) at the non-toxic dose (25 µM) significantly increased cell survival after infection of A549 cells with a pathogenic strain of TBEV (Hypr strain) (Figure 2). The antiviral activity of all glycoconjugates was further evaluated in the virus plaque formation assay, using a low pathogenic strain of TBEV (Neudoerfl strain) (Figure 3). Again, only these four compounds showed strong antiviral activity at the same dose, reducing the formation of viral plaques. Compounds **18** and **19** belong to the type II glycoconjugates, while compounds **20** and **21** to type III (Figure 1). It is worth noting that both active types of glycoconjugates contain a 1,2,3-triazole ring in the linker structure which results from the CuAAC reaction of glycosyl azides with propargyl derivatives of uridine. The presence of TBDMS groups in the uridine fragment turned out to be significant factor for the activity. This is evidenced by the significantly lower activity of glycoconjugates **16** and **17** containing isopropylidene protection in the uridine fragment, which represent the analogues of active compounds **18** and **19**. The presence of an amide moiety in glycoconjugates **18** and **19** also significantly affects the cytotoxicity of these compounds, which translates into a reduced selectivity index. When the amide bond in the uridine fragment was replaced with a linkage by an oxygen atom (compounds **20** and **21**), the cytotoxicity of the glycoconjugates thus obtained decreased two-fold, while their activity increased significantly. On the other hand, the type of sugar unit present in the glycoconjugates has a little effect on the observed activity and cytotoxicity.

Dose-dependent studies of the influence of four the most active compounds were performed using a plaque reduction assay (Figure 4). Moreover, the dose-dependent activity on TBEV titer reduction were used for the estimation of IC_50_ values (Figure 5, Table 1). Compound **20** showed the most potent antiviral activity, reaching 3.7 µM as IC_50_ value, which resulted in the highest SI of 49,7. The IC_50_ value of compound **21** was also high—4.7 µM. Two other compounds (**18** and **19**) demonstrated moderate level of TBEV propagation inhibition (IC_50_ = 9.3 and 15.1 µM, respectively). Our observations are similar to previously published data, that the lack of *N*-glycosylation site in the E protein of TBEV reduces virion secretion out of mammalian cells [17].

To further characterize the activity of tested compounds, we investigated their influence on the production of E protein using Western blot analysis, as tested compounds were designed to inhibit the activity of β-1,4-galactosyltransferase I during the late steps of *N*-glycosylation process. The production of E protein was affected by compounds studied by us in a dose-dependent manner. The production of viral proteins was greatly decreased at all tested concentrations as shown for the representative compound **21** (Figure 6). For the most active compound **20**, the complete inhibition of viral protein synthesis was observed at all tested doses of this compound. It is worthwhile to note that neither less glycosylated nor non-glycosylated forms of E protein were detected probably due to the quick degradation process of non-matured forms. The reduction in the glycoprotein E production after inhibitory treatment can be the reason for the decrease in TBEV viral production observed in previous experiments.

Summarizing, the four tested compounds (**18**, **19**, **20** and **21**) showed significant antiviral activity against two strains of tick-borne encephalitis virus. On the basis of these results, we conclude that compounds targeting *N*-glycosylation process of viral proteins may constitute a new promising approach in antiviral strategy.

## 4. Materials and Methods

### 4.1. General Information

Nuclear magnetic resonance (^1^H-NMR and ^13^C-NMR) spectra were determined in CDCl_3_ or DMSO-d6 using tetramethyl silane (TMS) as an internal standard and recorded with an Agilent spectrometer at a frequency of 400 MHz or with a Varian spectrometer at a frequency of 600 MHz. NMR solvents were purchased from ACROS Organics (Geel, Belgium). Chemical shifts (δ) are given in parts per million (ppm) and coupling constants (*J*) are given in Hz. Splitting patterns are designated as follows: s, singlet; d, doublet; dd, doublet of doublets; t, triplet; dd~t, doublet of doublets resembling a triplet; ddd, doublet of doublet of doublets; m, multiplet; bs, broad singlet. Optical rotations were measured with a JASCO P-2000 polarimeter using a sodium lamp (589.3 nm) at room temperature. Melting point measurements were performed on OptiMelt (MPA 100) Stanford Research Systems. Mass spectra were recorded with a WATERS LCT Premier XE LC/MS system (high-resolution mass spectrometer equipped with an electron spray ionization source and a high-resolution orthogonal TOF analyzer). Reactions were monitored by Thin-Layer Chromatography (TLC) on precoated plates of silica gel 60 F254 (Merck KGaA, Darmstadt, Germany). TLC plates were inspected under UV light (λ = 254 nm) or charring after spraying with 10% sulfuric acid in ethanol. Products were purified using column chromatography performed on Silica Gel 60 (70–230 mesh, Merck KGaA, Darmstadt, Germany) developed with toluene/EtOAc or CHCl_3_/MeOH solvent systems in various volume ratios. All evaporations were performed on a rotary evaporator under reduced pressure at 45 °C.

2,3,4,6-Tetra-*O*-acetyl-β-D-glucopyranosyl azide (**1**), 2,3,4,6-tetra-*O*-acetyl-β-D-galactopyranosyl azide (**2**) [23], 2,3,4,6-tetra-*O*-acetyl-β-D-glucopyranosyl amine (**3**), 2,3,4,6-tetra-*O*-acetyl-β-D-galactopyranosyl amine (**4**), 2,3,4,6-tetra-*O*-benzyl-β-D-galactopyranosyl amine (**5**) [31], propargyl 2,3,4,6-tetra-*O*-acetyl-β-D-glucopyranoside (**6**), propargyl 2,3,4,6-tetra-*O*-acetyl-β-D-galactopyranoside (**7**) [32], 2′,3′-*O*-isopropylidene-uridine-5′-carboxylic acid (**8**) [50], 5′-azido-5′-deoxy-2′,3′-*O*-isopropylidene-uridine (**9**) [51], *N*-propargyl-2′,3′-*O*-isopropylideneuridine-5′-amide (**10**) [24], 2′,3′-di-*O*-*tert*-butyldimethylsilyl-uridine-5′-carboxylic acid (**11a**) [18], 2′,3′-di-*O*-*tert*-butyldimethylsilyl-uridine (**12a**) [52] as well as glycoconjugates (**13**, **14**, **16** and **17**) [29] were prepared according to the respective published procedures. Sugars, uridine and other used chemicals were purchased from Sigma-Aldrich (Steinheim, Germany), ACROS Organics (Geel, Belgium) or Avantor (Gliwice, Poland) and were used without purification.

### 4.2. Chemistry

#### 4.2.1. Procedure for Synthesis of *N*-Propargyl-2′,3′-*O*-Tert-Butyldimethylsilyluridine-5′-Amide **11**

2′,3′-Di-*O*-*tert*-butyldimethylsilyl-uridine-5′-carboxylic acid **11a** (224 mg, 0.46 mmol) was dissolved in dichloromethane (6 mL) followed by addition of DCC (96 mg, 0.46 mmol) and propargyl amine (32 µL, 0.50 mmol) and mixed for 24 h at r.t. The reaction progress was monitored on TLC in an eluents system toluene:ethyl acetate (1:1). After the reaction was completed, the reaction mixture was concentrated under diminished pressure and the crude product was purified by column chromatography (toluene:ethyl acetate; gradient: 10:1 to 6:1) to give **11** (180 mg, 75%) as a colorless oil.

*N-propargyl-2′,3′-di-O-tert-butyldimethylsilyl-uridine-5′-amide* (**11**): ${\left[\alpha \right]}_{D}^{25}$ = −27.7 (c = 2.4, CHCl_3_), ^1^H-NMR (CDCl_3_, 400 MHz) δ: −0.02, 0.04, 0.12, 0.18 (4s, 12H, CH_3_Si), 0.85, 0.93 (2s, 6H, (CH_3_)_3_C), 2.23 (dd~t, 1H, *J* = 2.4 Hz, *J* = 2.4 Hz, CH_2_CCH), 4.02–4.15 (m, 2H, CH_2_CCH), 4.26 (dd, 1H, *J* = 1.2 Hz, *J* = 4.3 Hz, H-3′), 4.39 (d, 1H, *J* = 1.2 Hz, H-4′), 4.68 (dd, 1H, *J* = 4.3 Hz, *J* = 7.2 Hz, H-2′), 5.39 (d, 1H, *J* = 7.2 Hz, H-1′), 5.78 (d, 1H, *J* = 8.0 Hz, H-5_ur_), 7.44 (d, 1H, *J* = 8.0 Hz, H-6_ur_), 7.77 (t, 1H, *J* = 5.1 Hz, NHCH_2_). ^13^C-NMR (CDCl_3_, 100 MHz) δ: −5.13, −4.74, −4.61 (CH_3_Si), 17.85, 17.99 ((CH_3_)_3_C), 25.67, 25.76 ((CH_3_)_3_C), 28.80 (NHCH_2_CCH), 70.65, 71.44, 74.73 (NHCH_2_CCH, C-2′, C-3′), 79.03 (NHCH_2_CCH), 85.24(C-4′), 95.34 (C-1′), 102.77 (C-5_ur_), 144.41 (C-6_ur_), 150.38 (C-2_ur_), 162.08 (C-4_ur_), 169.09 (CONH). ESI-HRMS: calcd for C_24_H_41_N_3_O_6_Si_2_Na [M + Na]^+^: *m/z* 546.2432. Found: *m/z* 546.2435.

#### 4.2.2. Procedure for Synthesis Of 2′,3′-Di-O-Tert-Butyldimethylsilyl-5′-*O*-Propargyl-Uridine **12**

2′,3′-Di-*O*-*tert*-butyldimethylsilyluridine **12a** (482 mg, 1.03 mmol) was dissolved in THF (10 mL) followed by addition of sodium hydride (130 mg, 5.42 mmol). The flask with the reaction mixture was placed in an ice bath and stirred for 30 min. Next propargyl bromide (102 µL, 1.18 mmol) was added and the whole mixture was placed in an ultrasonic bath for 4 h. The reaction progress was monitored on TLC in an eluents system toluene:ethyl acetate (1:1). After the reaction was completed, methanol (1 mL) and water (5 mL) were added to the reaction mixture. The product was extracted with ethyl acetate (3 × 10 mL). The combined organic phases were dried over anhydrous MgSO_4_, filtered and concentrated under reduced pressure. The crude product was purified by column chromatography (toluene:ethyl acetate; gradient: 20:1 to 8:1) to give **12** (309 mg, 59%) as a white solid.

*2′,3′-Di-O-tert-butyldimethylsilyl-5′-O-propargyl-uridine* (**12**): ${\left[\alpha \right]}_{D}^{25}$ = 37.6 (c = 1.0, CHCl_3_), *m.p.* = 66–68 °C, ^1^H-NMR (CDCl_3_, 400 MHz) δ: 0.08, 0.09, 0.12, 0.13 (4s, 12H, CH_3_Si), 0.90, 0.91 (2s, 18H, (CH_3_)_3_C), 2.49 (dd~t, 1H, *J* = 2.4 Hz, *J* = 2.4 Hz, CH_2_CCH), 3.68 (dd, 1H, *J* = 2.0 Hz, *J* = 10.6 Hz, H-5′b), 3.87 (dd, 1H, *J* = 2.2 Hz, *J* = 10.6 Hz, H-5′a), 4.08–4.18 (m, 3H, H-2′, H-3′, H-4′), 3.96–4.27 (m, 2H, CH_2_CCH), 4.16 (ddd, 1H, *J* = 2.6 Hz, *J* = 6.3 Hz, *J* = 17.6 Hz, NHCH_2_CCH), 5.70 (dd, 1H, *J* = 2.2 Hz, *J* = 8.0 Hz, H-5_ur_), 5.79 (d, 1H, *J* = 3.1 Hz, H-1′), 8.00 (d, 1H, *J* = 8.2 Hz, H-6_ur_), 8.62 (s, 1H, NH). ^13^C-NMR (CDCl_3_, 100 MHz) δ: −5.01, −4.86, −4.57, −4.30 (CH_3_Si), 18.01, 18.07 ((CH_3_)_3_C), 25.79, 25.83 ((CH_3_)_3_C), 58.58 (OCH_2_CCH), 67.96 (C-5′), 70.70, 75.15, 75.91 (C-2′, C-3′, CH_2_CCH), 78.68 (CH_2_CCH), 82.59 (C-4′), 89.60 (C-1)’, 101.69 (C-5_ur_), 140.48 (C-6_ur_), 150.19 (C-2_ur_), 163.20 (C-4_ur_). ESI-HRMS: calcd for C_24_H_42_N_2_O_6_Si_2_Na [M + Na]^+^: *m/z* 533.2479. Found: *m/z* 533.2478.

#### 4.2.3. General Procedure for Synthesis of **Glycoconjugates 13–15** By Amide Bond Formation

2′,3′-*O*-Isopropylidene-uridine-5′-carboxylic acid **8** (86 mg, 0.29 mmol) was dissolved in MeOH (3 mL) followed by addition of acetylated glycosyl amine **3** or **4** (100 mg, 0.29 mmol) or benzylated glycosyl amine **5** (156 mg, 0.29 mmol) and mixed for 5 min at r.t. Then, 2-chloro-4,6-dimethoxy-1,3,5-triazine (56 mg, 0.32 mmol) and 4-methylmorpholine (35 μL, 0.32 mmol) were added and the reaction was continued for 5 h at r.t. The reaction mixture was concentrated under reduced pressure, dissolved in CH_2_Cl_2_ (30 mL) and washed with brine (5 mL), saturated NaHCO_3_ (5 mL) and once again with brine (5 mL). The organic layer was dried over anhydrous MgSO_4_ and concentrated under reduced pressure. Crude products were purified by column chromatography (chloroform:methanol; gradient: 100:1 to 75:1 in case of products **13** and **14** or toluene:ethyl acetate; gradient 4:1 to 2:1 in case of product **15**) to give **13** (144 mg, 80%) or **14** (155 mg, 86%) or **15** (100 mg, 42%) as white solids.

*N-(2″,3″,4″,6″-tetra-O-benzyl-β-D-galactopyranosyl)-2′,3′-O-isopropylidene-uridine-5′-amide* (**Glycoconjugate 15**): ${\left[\alpha \right]}_{D}^{20}$ = −17.3 (c = 1.0, CHCl_3_), ^1^H-NMR (CDCl_3_, 400 MHz) δ: 1.21, 1.53 (2s, 6H, (CH_3_)_2_C), 3.52–3.59 (m, 2H, H-6a_gal_, H-6b_gal_), 3.68 (dd, 1H, *J* = 2.4 Hz, *J* = 9.4 Hz, H-3_gal_), 3.69 (m, 1H, H-5_gal_), 3.82 (dd~t, 1H, *J* = 9.0 Hz, *J* = 9.4 Hz, H-2_gal_), 3.99 (d, 1H, *J* = 2.4 Hz, H-4_gal_), 4.40 and 4.45 (qAB, 2H, *J* = 12.0 Hz, CH_2_Ph), 4.43 (d, 1H, *J* = 3.2 Hz, H-4′), 4.59 and 4.91 (qAB, 2H, *J* = 11.3 Hz, CH_2_Ph), 4.66 and 4.73 (qAB, 2H, *J* = 11.6 Hz, CH_2_Ph), 4.75 and 4.92 (qAB, 2H, *J* = 11.7 Hz, CH_2_Ph), 4.77 (dd, 1H, *J* = 3.1 Hz, *J* = 6.6 Hz, H-2′), 4.82 (dd, 1H, *J* = 3.2 Hz, *J* = 6.6 Hz, H-3′), 5.20 (dd~t, 1H, *J* = 9.0 Hz, *J* = 9.4 Hz, H-1_gal_), 5.43 (dd, 1H, *J* = 3.1 Hz, H-1′), 5.62 (dd, 1H, *J* = 1.9 Hz, *J* = 7.8 Hz, H-5_ur_), 7.09 (d, 1H, *J* = 7.8 Hz, H-6_ur_), 7.20 (d, 1H, *J* = 9.4 Hz, NH_amide_), 7.23–7.38 (m, 20H, H-Ph), 8.22 (bs, 1H, NH). ^13^C-NMR (CDCl_3_, 100 MHz) δ: 27.14, 25.02 ((CH_3_)_2_C), 68.10 (C-6_gal_), 72.54, 73.45, 73.46, 74.16 (CH_2_Ph), 74.80, 75.01 (C-4_gal_, C-5_gal_), 78.04 (C-3′), 78.98, 82.07 (C-2_gal_, C-3_gal_), 82.67 (C-2′), 83.29 (C-1_gal_), 85.31 (C-4′), 96. 86 (C-1′), 102.91 (C-5_ur_), 127.13, 127.41, 127.47, 127.63, 127.73, 127.76, 127.81, 127.86, 127.94, 127.99, 128.22, 128.28, 128.3, 128.35, 128.41, 128.44, 137.73, 138.01, 138.40, 138.78 (C-Ph), 142.78, 149.81 (C-6ur, C-2_ur_), 162.20 (C-4_ur_), 169,08 (CONH).ESI-HRMS: calcd for C_46_H_49_N_3_O_11_Na [M + Na]^+^: *m/z* 842.3265. Found: *m/z* 842.3268.

#### 4.2.4. General Procedure for Synthesis of **Glycoconjugates 16–23** by CuAAC Reaction

Procedure **A**: To a solution of *O*-acetylated glycosyl azides **5** or **6** (86 mg, 0.23 mmol) and *N*-propargyl-2′,3′-*O*-isopropylidene-uridine-5′-amide **10** (77 mg, 0.23 mmol) or *N*-propargyl-2′,3′-di-*O*-*tert*-butyldimethylsilyl-uridine-5′-amide **11** (120 mg, 0.23 mmol) or 2′,3′-di-*O*-*tert*-butyldimethylsilyl-5′-*O*-propargyl-uridine **12** (117 mg, 0.23 mmol) in THF (2 mL) and *i-*PrOH (2 mL) mixture, CuSO_4_·5H_2_O (12 mg, 0.046 mmol) dissolved in H_2_O (1 mL) was added followed by addition of sodium ascorbate (18 mg, 0.09 mmol) dissolved in H_2_O (1 mL). The reaction mixture was stirred for 5 h (during synthesis of products **16** and **17**) or for 24 h at r.t. (synthesis of products **18–23**). Mixture was concentrated in vacuo and purified by column chromatography to give products **16–23**.

*1-(2″,3″,4″,6″-tetra-O-acetyl-β-D-glucopyranosyl)-4-(2′,3′-di-O-tert-butyldimethylsilyl-uridine-5′-carbonylaminomethyl)-[1,2,3]-triazole* (**Glycoconjugate 18**): The crude product was purified by column chromatography (toluene:ethyl acetate; gradient: 6:1 to 1:1) to give **18** (142 mg, 69%) as a white solid. ${\left[\alpha \right]}_{D}^{25}$ = −32.6 (c = 1.0, CHCl_3_), m.p. = 117–120 °C, ^1^H-NMR (CDCl_3_, 400 MHz) δ: -0.04, 0.04, 0.13, 0.19 (4s, 12H, CH_3_Si), 0.84, 0.94 (2s, 18H, (CH_3_)_3_C), 1.90, 2.04, 2.07, 2.09 (4s, 12H, CH_3_CO), 4.01 (ddd, 1H, *J* = 2.0 Hz, *J* = 4.4 Hz, *J* = 9.8 Hz, H-5_glu_), 4.17 (dd, 1H, *J* = 2.0 Hz, *J* = 12.6 Hz, H-6a_glu_), 4.31–4.36 (m, 2H, H-6b_glu_, H-3′), 4.42 (s, 1H, H-4′), 4.52–4.70 (m, 2H, CH_2_), 4.71 (dd, 1H, *J* = 4.3 Hz, *J* = 7.8 Hz, H-2′), 5.23 (m, 1H, H-4_glu_), 5.37–5.45 (m, 3H, H-1′, H-2_glu_, H-3_glu_), 5.77 (dd, 1H, *J* = 2.0 Hz, *J* = 8.0 Hz, H-5_ur_), 5.88 (m, 1H, H-1_glu_), 7.41 (d, 1H, *J* = 8.0 Hz, H-6_ur_), 7.78 (s, 1H, H-5_triaz_), 8.08 (t, 1H, *J* = 5.1 Hz, NHCH_2_), 9.05 (s, 1H, NH). ^13^C-NMR (CDCl_3_, 100 MHz) δ: −5.17, −4.74, −4.71, −4.55 (CH_3_Si), 17.83, 17.98 ((CH_3_)_3_C), 20.24, 20.48, 20.50, 20.65 (CH_3_CO), 25.66, 25.76 ((CH_3_)_3_C), 34.99 (NHCH_2_), 61.42 (C-6_glu_), 67.61, 70.59, 70.61, 72.70, 74.76, 75.19 (C-4_glu_, C-3_glu_, C-2_glu_, C-5_glu_, C-2′, C-3′), 85.56, 85.73 (C-4′, C-1_glu_), 95.02 (C-1′), 102.89 (C-5_ur_), 120.11 (C-5_triaz._), 144.28, 145.04 (C-6_ur_, C-4_triaz._), 150.48 (C-2_ur_), 162.50 (C-4_ur_), 169.30, 169.36, 169.94, 170.52 (CO). ESI-LRMS: calcd for [M + Na]^+^: *m*/*z* 919.3553. Found: *m*/*z* 919.3560.

*1-(2″,3″,4″,6″-tetra-O-acetyl-β-D-galactopyranosyl)-4-(2′,3′-di-O-tert-butyldimethylsilyl-uridine-5′-carbonylaminomethyl)-[1,2,3]-triazole* (**Glycoconjugate 19**): The crude product was purified by column chromatography (toluene:ethyl acetate; gradient: 8:1 to 1:1) to give **19** (148 mg, 72%) as a white solid. ${\left[\alpha \right]}_{D}^{25}$ = −16.4 (c = 1.0, CHCl_3_), m.p. = 126–128 °C, ^1^H-NMR (CDCl_3_, 400 MHz) δ: -0.05, 0.04, 0.13, 0.20 (4s, 12H, CH_3_Si), 0.84, 0.94 (2s, 18H, (CH_3_)_3_C), 1.91, 2.02, 2.05, 2.22 (4s, 12H, CH_3_CO), 4.16–4.26 (m, 3H, H-5_gal_, H-6a_gal_, H-6b_gal_), 4.31 (dd, 1H, *J* = 0.8 Hz, *J* = 4.3 Hz, H-3′), 4.42 (d, 1H, *J* = 0.8 Hz, H-4′), 4.52 (m, 1H, CH), 4.66–4.74 (m, 2H, CH, H-2′), 5.24 (dd, 1H, *J* = 3.5 Hz, *J* = 10.0 Hz, H-3_gal_), 5.42 (d, 1H, *J* = 7.8 Hz, H-1′), 5.53 (dd~t, 1H, *J* = 9.4 Hz, *J* = 10.0 Hz, H-2_gal_), 5.55 (d, 1H, *J* = 3.5 Hz, H-4_gal_), 5.76 (dd, 1H, *J* = 2.3 Hz, *J* = 8.0 Hz, H-5_ur_), 5.84 (d, 1H, *J* = 9.4 Hz, H-1_gal_), 7.42 (d, 1H, *J* = 8.0 Hz, H-6_ur_), 7.83 (s, 1H, H-5_triaz_), 8.04 (t, 1H, *J* = 5.3 Hz, NHCH_2_), 8.85 (s, 1H, NH). ^13^C-NMR (CDCl_3_, 100 MHz) δ: −5.14, −4.69, −4.67, −4.51 (CH_3_Si), 17.87, 18.04 ((CH_3_)_3_C), 20.37, 20.50, 20.63, 20.65 (CH_3_CO), 25.69, 25.80 ((CH_3_)_3_C), 35.00 (NHCH_2_), 61.21 (C-6_gal_), 66.88 (C-4_gal_), 68.16 (C-3′), 70.69 (C-3_gal_), 70.85 (C-2_gal_), 74.21 (C-5_gal_), 74.81 (C-2′), 85.62, 86.30 (C-4′, C-1_gal_), 94.98 (C-1′), 102.87 (C-5_ur_), 120.32 (C-5_triaz._), 144.26, 144.75 (C-6_ur_, C-4_triaz._), 150.42 (C-2_ur_), 162.38 (C-4_ur_), 169.36, 169.55, 169.94, 170.36 (CO). ESI-LRMS: calcd for [M + Na]^+^: *m*/*z* 919.3553. Found: *m*/*z* 919.3563.

*1-(2″,3″,4″,6″-tetra-O-acetyl-β-D-glucopyranosyl)-4-(2′,3′-di-O-tert-butyldimethylsilyl-uridine-5′-O-methyl)-[1,2,3]-triazole* (**Glycoconjugate 20**): The crude product was purified by column chromatography (toluene:ethyl acetate; gradient: 6:1 to 2:1) to give **20** (146 mg, 72%) as a white solid. ${\left[\alpha \right]}_{D}^{25}$ = 4.0 (c = 1.0, CHCl_3_), m.p. = 105–108 °C, ^1^H-NMR (CDCl_3_, 400 MHz) δ: −0.01, 0.06, 0.07, 0.08 (4s, 12H, CH_3_Si), 0.88, 0.90 (2s, 18H, (CH_3_)_3_C), 1.87, 2.03, 2.04, 2.07 (4s, 12H, CH_3_CO), 3.68 (dd, 1H, *J* = 1.4 Hz, *J* = 10.6 Hz, H-5′a), 4.03 (ddd, 1H, *J* = 1.8 Hz, *J* = 4.9 Hz, *J* = 10.1 Hz, H-5_glu_), 4.09–4.15 (m, 3H, H-2′, H-3′, H-4′), 4.18 (dd, 1H, *J* = 1.8 Hz, *J* = 12.7 Hz, H-6a_glu_), 4.35 (dd, 1H, *J* = 4.9 Hz, *J* = 12.7 Hz, H-6b_glu_), 4.64 and 4.72 (qAB, 2H, *J* = 11.9 Hz, CH_2_), 5.25 (dd~t, 1H, *J* = 9.4 Hz, *J* = 10.1 Hz, H-4_glu_), 5.38 (dd~t, 1H, *J* = 9.0 Hz, *J* = 9.8 Hz, H-2_glu_), 5.43 (dd~t, 1H, *J* = 9.4 Hz, *J* = 9.8 Hz, H-3_glu_), 5.65 (dd, 1H, *J* = 1.8 Hz, *J* = 8.2 Hz, H-5_ur_), 5.78 (d, 1H, *J* = 3.1 Hz, H-1′), 5.89 (d, 1H, *J* = 9.0 Hz, H-1_glu_), 7.75 (s, 1H, H-5_triaz_), 7.94 (s, 1H, NH), 7.95 (d, 1H, *J* = 8.2 Hz, H-6_ur_). ^13^C-NMR (CDCl_3_, 100 MHz) δ: −4.98, −4.82, −4.62, −4.32 (CH_3_Si), 18.01, 18.10 ((CH_3_)_3_C), 20.13, 20.50, 20.54, 20.68 (CH_3_CO), 25.80, 25.84 ((CH_3_)_3_C), 61.54 (C-6_glu_), 64.32 (CH_2_), 67.75 (C-5′), 68.60, 70.39 (C-3_glu_, C-4_glu_), 70.94, 72.51, 75.41, 75.84 (C-2_glu_, C-5_glu_, C-2′, C-3′), 82.83, 85.91 (C-4′, C-1_glu_), 89.46 (C-1′), 101.86 (C-5_ur_), 120.60 (C-5_triaz._), 140.75, 144.71 (C-6_ur_, C-4_triaz._), 150.11 (C-2_ur_), 162.75 (C-4_ur_), 169.02, 169.37, 169.80, 170.39 (CO). ESI-LRMS: calcd for [M + Na]^+^: *m*/*z* 906.3600. Found: *m*/*z* 906.3581.

*1-(2″,3″,4″,6″-tetra-O-acetyl-β-D-galactopyranosyl)-4-(2′,3′-di-O-tert-butyldimethylsilyl-uridine-5′-O-methyl)-[1,2,3]-triazole* (**Glycoconjugate 21**): The crude product was purified by column chromatography (toluene:ethyl acetate; gradient: 6:1 to 2:1) to give **21** (150 mg, 74%) as a white solid. ${\left[\alpha \right]}_{D}^{25}$ = 17.2 (c = 1.0, CHCl_3_), m.p. = 104–107 °C, ^1^H-NMR (CDCl_3_, 400 MHz) δ: 0.05, 0.06, 0.07, 0.08 (4s, 12H, CH_3_Si), 0.88, 0.90 (2s, 18H, (CH_3_)_3_C), 1.88, 2.02, 2.04, 2.21 (4s, 12H, CH_3_CO), 3.68 (dd, 1H, *J* = 2.2 Hz, *J* = 10.8 Hz, H-5′a), 3.88 (dd, 1H, *J* = 2.2 Hz, *J* = 10.8 Hz, H-5′b), 4.09–4.27 (m, 6H, H-5_gal_, H-6a_gal_, H-6b_gal_, H-2′, H-3′, H-4′), 4.65 and 4.73 (qAB, 2H, *J* = 11.9 Hz, CH_2_), 5.27 (dd, 1H, *J* = 3.5 Hz, *J* = 10.2 Hz, H-3_gal_), 5.50 (dd~t, 1H, *J* = 9.4 Hz, *J* = 10.2 Hz, H-2_gal_), 5.57 (d, 1H, *J* = 3.5 Hz, H-4_gal_), 5.66 (dd, 1H, *J* = 2.4 Hz, *J* = 8.2 Hz, H-5_ur_), 5.79 (d, 1H, *J* = 3.1 Hz, H-1′), 5.86 (d, 1H, *J* = 9.4 Hz, H-1_gal_), 7.80 (s, 1H, H-5_triaz_), 7.95 (d, 1H, *J* = 8.2 Hz, H-6_ur_), 8.27 (s, 1H, NH). ^13^C-NMR (CDCl_3_, 100 MHz) δ: −4.84, −4.69, −4.47, −4.19 (CH_3_Si), 18.14, 18.24 ((CH_3_)_3_C), 20.36, 20.61, 20.73, 20.76 (CH_3_CO), 25.93, 25.98 ((CH_3_)_3_C), 61.14 (C-6_gal_), 64.51 (CH_2_), 66.91, 68.18, 68.75, 70.76, 71.10, 74.36, 76.00 (C-5′, C-4gal, C-3_gal_, C-3′, C-2_gal_, C-5_gal_, C-2′), 82.92, 86.55 (C-4′, C-1_gal_), 89.54 (C-1′), 101.99 (C-5_ur_), 120.85 (C-5_triaz._), 140.84, 144.78 (C-6_ur_, C-4_triaz._), 150.32 (C-2_ur_), 163.07 (C-4_ur_), 169.36, 169.88, 170.03, 170.44 (CO). ESI-LRMS: calcd for [M + Na]^+^: *m*/*z* 906.3600. Found: *m*/*z* 906.3604.

*2′,3′-O-isopropylidene-5′-deoxy-5′-[4-(2″,3″,4″,6″-tetra-O-acetyl-β-D-glucopyranosyl-oxymethyl)-1,2-3-triazol-1-yl]uridine* (**Glycoconjugate 22**): The crude product was purified by column chromatography (toluene:ethyl acetate; gradient: 15:1 to 2:1 and next chloroform:methanol; gradient 100:1 to 40:1) to give **22** (142 mg, 89%) as a white solid. ${\left[\alpha \right]}_{D}^{24}$ = 7.3 (c = 1.0, CHCl_3_), m.p. = 108–110 °C, ^1^H-NMR (CDCl_3_, 400 MHz) δ: 1.33, 1.55 (2s, 6H, (CH_3_)_2_C), 1.99, 2.00, 2.03, 2.09 (4s, 12H, CH_3_CO), 3.73 (ddd, 1H, *J* = 2.3 Hz, *J* = 4.9 Hz, *J* = 9.8 Hz, H-5_glu_), 4.15 (dd, 1H, *J* = 2.3 Hz, *J* = 12.1 Hz, H-6a_glu_), 4.23 (dd, 1H, *J* = 4.9 Hz, *J* = 12.7 Hz, H-6b_glu_), 4.47 (ddd, 1H, *J* = 4.7 Hz, *J* = 6.6 Hz, *J* = 6.8 Hz, H-4′), 4.67 (d, 1H, *J* = 8.2 Hz, H-1_glu_), 4.68 (dd, 1H, *J* = 6.8 Hz, *J* = 14.1 Hz, H-5′a), 4.73 (dd, 1H, *J* = 4.3 Hz, *J* = 14.1 Hz, H-5′b), 4.81 and 4.93 (qAB, 2H, *J* = 12.5 Hz, CH_2_), 4.91 (dd, 1H, *J* = 4.7 Hz, *J* = 6.7 Hz, H-3′), 5.10 (dd, 1H, *J* = 8.2 Hz, *J* = 9.4 Hz, H-2_glu_), 5.17 (dd, 1H, *J* = 2.0 Hz, *J* = 6.7 Hz, H-2′), 5.19 (dd~t, 1H, *J* = 9.4 Hz, *J* = 9.8 Hz, H-4_glu_), 5.21 (dd~t, 1H, *J* = 9.4 Hz, *J* = 9.4 Hz, H-3_glu_), 5.50 (d, 1H, *J* = 2.0 Hz, H-1′), 5.75 (dd, 1H, *J* = 2.3 Hz, *J* = 7.8 Hz, H-5_ur_), 7.08 (d, 1H, *J* = 7.8 Hz, H-6_ur_), 7.56 (s, 1H, H-5_triaz_), 8.59 (s, 1H, NH). ^13^C-NMR (CDCl_3_, 100 MHz) δ: 20.62, 20.70, 20.77 (CH_3_CO), 25.28, 27.13 (CH_3_)_2_C), 51.64 (C-5′), 61.92, 63.16 (C-6_glu_, CH_2_), 68.42, 71.28, 71.97, 72.67 (C-2_glu_, C-3_glu_, C-4_glu_, C-5_glu_), 81.54, 84.03, 85.92 (C-2′, C-3′, C-4′), 96.33 (C-1′), 100.23 (C-1_glu_), 103.01 (C-5_ur_), 114.96 (CH_3_)_2_C), 124.29 (C-5_triaz._), 143.22, 144.11 (C-6_ur_, C-4_triaz._), 149.75 (C-2_ur_), 162.48 (C-4_ur_), 169.56, 170.24, 170.74 (CO). ESI-LRMS: calcd for [M + H]^+^: *m*/*z* 696.2364. Found: *m*/*z* 696.2407.

*2′,3′-O-isopropylidene-5′-deoxy-5′-[4-(2″,3″,4″,6″-tetra-O-acetyl-β-D-galactopyranosyl-oxymethyl)-1,2-3-triazol-1-yl]uridine* (**Glycoconjugate 23**): The crude product was purified by column chromatography (toluene:ethyl acetate; gradient: 15:1 to 2:1 and next chloroform:methanol; gradient 100:1 to 40:1) to give **23** (104 mg, 65%) as a white solid. ${\left[\alpha \right]}_{D}^{24}$ = 1.2 (c = 1.0, CHCl_3_), m.p. = 101–105 °C, ^1^H-NMR (CDCl_3_, 400 MHz) δ: 1.34, 1.55 (2s, 6H, (CH_3_)_2_C), 1.98, 2.00, 2.06, 2.15 (4s, 12H, CH_3_CO), 3.95 (ddd, 1H, *J* = 1.0 Hz, *J* = 6.5 Hz, *J* = 6.8 Hz, H-5_gal_), 4.13 (dd, 1H, *J* = 6.5 Hz, *J* = 11.2 Hz, H-6a_gal_), 4.17 (dd, 1H, *J* = 6.8 Hz, *J* = 11.2 Hz, H-6b_gal_), 4.46 (m, 1H, H-4′), 4.65 (d, 1H, *J* = 8.2 Hz, H-1_gal_), 4.80 and 4.97 (qAB, 2H, *J* = 12.5 Hz, CH_2_), 4.68–4.77 (m, 2H, H-5′a, H-5′b), 4.87 (dd, 1H, *J* = 4.7 Hz, *J* = 6.8 Hz, H-3′), 5.02 (dd, 1H, *J* = 3.5 Hz, *J* = 10.2 Hz, H-3_gal_), 5.06 (dd, 1H, *J* = 2.0 Hz, *J* = 6.8 Hz, H-2′), 5.21 (dd, 1H, *J* = 8.2 Hz, *J* = 10.2 Hz, H-2_gal_), 5.90 (dd, 1H, *J* = 1.0 Hz, *J* = 3.5 Hz, H-4_gal_), 5.55 (d, 1H, *J* = 2.0 Hz, H-1′),5.79 (dd, 1H, *J* = 2.0 Hz, *J* = 7.8 Hz, H-5_ur_), 7.11 (d, 1H, *J* = 7.8 Hz, H-6_ur_), 7.58 (s, 1H, H-5_triaz_), 9.20 (s, 1H, NH). ^13^C-NMR (CDCl_3_, 100 MHz) δ: 20.56, 20.64, 20.70, 20.80 (CH_3_CO), 25.24, 27.10 (CH_3_)_2_C), 51.60(C-5′), 61.31, 63.22 (CH_2_, C-6_gal_), 67.04, 68.88, 70.75, 70.86 (C-2_gal_, C-3_gal_, C-4_gal_, C-5_gal_), 81.39, 83.91, 85.74 (C-2′, C-3′, C-4′), 95.86 (C-1′), 100.82 (C-1_gal_), 103.05 (C-5_ur_), 115.00 (CH_3_)_2_C), 124.34 (C-5_triaz._), 143.24, 144.10 (C-6_ur_, C-4_triaz._), 150.01 (C-2_ur_), 162.92 (C-4_ur_), 169.73, 170.11, 170.29, 170.49 (CO). ESI-LRMS: calcd for [M + H]^+^: *m*/*z* 696.2364. Found: *m*/*z* 696.2468.

### 4.3. Biological Evaluation of Compounds Activity

#### 4.3.1. Antiviral Compounds

The synthesized compounds were dissolved in DMSO and stored at −20°C until future use.

#### 4.3.2. Cells and Viruses

The A549 cell line (adenocarcinomic human alveolar basal epithelial cells, ATCC^®^ CCL-185™) was cultured in Dulbecco’s Modified Eagle’s Medium (D-MEM) (Corning, New York, USA) supplemented with 8% heat-inactivated fetal bovine serum (FBS), 100 U/mL penicillin, and 100 g/mL streptomycin at 37 °C under 5% CO_2_. The Neudoerfl TBEV strain, kindly provided by Dr. Karin Stiasny (Center for Virology, Medical University of Vienna, Vienna, Austria) and the Hypr TBEV strain, kindly provided by Dr. Daniel Růžek (Department of Virology, Veterinary Research Institute, Brno, Czech Republic) were cultured in A549 cells for 3–4 days. The plaque assay method was used to determine TBEV virus titers.

#### 4.3.3. Cell Viability Assay

A549 cell viability and compound cytotoxicity were estimated by the CellTiter 96 AQ_ueous_ non-radioactive cell proliferation assay (MTS) (Promega, Madison, WI, USA) according to the manufacturer’s instructions. The cytotoxicity measurements were done in three replicates. The half-maximal cytotoxic concentration (CC_50_) values of compounds that are cytotoxic in 50% were calculated using the GraphPad Prism software (version 5.01, GraphPad Software, San Diego, CA, USA) from the dose–response curves.

#### 4.3.4. CPE Inhibition Assay

A549 cells were seeded together with different doses of tested compounds or DMSO (positive control) and infected with TBEV (Hypr strain; multiplicity of infection (MOI) of 0.1) for 96 h. CPE was measured by the colorimetric CytoTox 96^®^ Non-Radioactive Cytotoxicity Assay (Promega, Madison, WI, USA) according to the manufacturer’s instructions. One-way ANOVA with Dunnett’s post-test was performed for statistical analysis using the GraphPad Prism software (version 5.01, GraphPad Software, San Diego, CA, USA).

#### 4.3.5. Plaque Assay

Monolayers of A549 cells grown in 24-well culture plates were inoculated with 10-fold dilutions of TBEV strains for 2 h at 37 °C. After the virus was removed, the cells were overlaid with carboxymethylcellulose in Modified Eagle’s Medium (MEM) medium. After 5 days the medium was removed, the cells were washed with PBS and stained with the solution of 0.005% amido black to count the plaque-forming units (PFU). The virus titers were expressed in terms of PFU/mL.

#### 4.3.6. Plaque Reduction Assay

Monolayers of A549 cells seeded in 12-well plates were inoculated with TBEV at MOI of 0.001. Virus inoculum was removed after 2 h at 37 °C, the cells were washed and overlaid with 1% carboxymethylcellulose in Modified Eagle’s Medium (MEM) medium with DMSO or tested compounds. After three days, cells were washed with phosphate-buffered saline (PBS) and fixed with methanol. The infected virus foci were visualized by immunostaining with the monoclonal anti-Flavivirus group antigen antibody (4G2) (Absolute Antibody, Oxford, UK; diluted 1:1500 in PBS, 0,05% Tween 80, 5% FBS) as a primary antibody and anti-mouse secondary antibody conjugated with horseradish peroxidase (HRP) (diluted 1:2000) in PBS containing 0,05% Tween 80 and 5% FBS. Plaques were detected using a Vector Nova Red kit (Vector Laboratories Ltd., Peterborough, UK) according to the manufacturer’s protocol and counted. If necessary, one-way ANOVA with Dunnett’s post-test was performed in some experiments for statistical analysis using the GraphPad Prism software (version 5.01, GraphPad Software, San Diego, CA, USA).

#### 4.3.7. Dose-Response Antiviral Activity of Tested Compounds

A549 cells were infected with TBEV Neudoerfl strain (MOI = 0.1) and treated with different concentrations of the tested compounds (0 to 25 µM). Culture supernatants were collected two days post-infection and used for the estimation of viral titers using the plaque assay. The dose–response curves were prepared using GraphPad Prism software and used for calculations of the half-maximal inhibitory concentration (IC_50_) values, indicating the concentration required to reduce the viral titer by 50% compared to the non-treated virus infected cells.

#### 4.3.8. Western Blot Analysis

A monolayer of A549 cells in 12-well plates was inoculated with TBEV at an MOI of 0.1 for 2 h. The virus was collected and fresh medium with different concentrations of compounds or DMSO was added and cultured for further 48 h at 37 °C. Cells were lysed at 4 °C for 1 h with TNET buffer (20 mM Tris–HCl (pH 7.4), 150 mM NaCl, 1 mM EDTA, 1% Triton X-100). Total concentration of proteins in lysates was measured using a Quick Start™ Bradford Protein Assay (Bio-Rad) according to the manufacturer’s protocol. An amount of 50 µg of proteins of each lysate were separated on a 10–20% SDS-PAGE gel under denaturing and reducing conditions, transferred to PVDF membranes, and detected with a specific monoclonal anti-Flavivirus group antigen antibody (4G2) (1:1500 dilution) followed by anti-mouse peroxidase (HRP)-conjugated secondary antibodies (diluted 1:2000). Blots were developed using a Super Signal™ West Pico Plus Substrate system (Pierce, Dallas, TX, USA) using Chemidoc system Alliance™ Q9-Series (UVITEC, Cambrige, United Kingdom).

## Figures and Tables

**Table 1:** Antiviral activity of uridine glycoconjugates **18–21** against TBEV Neudoerfl strain.

Type of Uridine Glycoconjugate Structure	Compound	CC_50_ (µM) ^a^	IC_50_ (µM) ^b^	SI ^c^
**II**	**18**	69	9.3	7.4
	**19**	77	15.1	5.1
**III**	**20**	184	3.7	49.7
	**21**	116	4.7	24.7

^a^ Concentration of the compound causing reduction in cell viability by 50%; ^b^ Concentration to reduce infectious viral titers by 50%; ^c^ Selectivity index (SI): CC_50_/IC_50_.
